# 7-Chloro-1,5-dipropargyl-1*H*-1,5-benzodiazepine-2,4(3*H*,5*H*)-dione

**DOI:** 10.1107/S1600536810047008

**Published:** 2010-11-20

**Authors:** Rachid Dardouri, Youssef Kandri Rodi, Natalie Saffon, El Mokhtar Essassi, Seik Weng Ng

**Affiliations:** a Laboratoire de Chimie Organique Hétérocyclique, Pôle de Compétences Pharmacochimie, Université Mohammed V-Agdal, BP 1014 Avenue Ibn Batout, Rabat, Morocco; b Service Commun Rayons-X FR2599, Université Paul Sabatier, Bâtiment 2R1, 118 route de Narbonne, Toulouse, France; c Department of Chemistry, University of Malaya, 50603 Kuala Lumpur, Malaysia

## Abstract

The seven-membered ring of the title compound, C_15_H_11_ClN_2_O_2_, adopts a boat-shaped conformation (with the C atoms of the fused-ring as the stern and the methyl­ene C atom as the prow). The N atoms exists in a trigonal–planar coordination; one of the acetyl­enic H atoms forms a C—H⋯O hydrogen bond to the O atom of an adjacent mol­ecule, generating a linear chain along a body diagonal.

## Related literature

For the crystal structure of 1,5-dimethyl-1,5-benzodiazepin-2,4-dione, see: Mondieig *et al.* (2005 ▶).
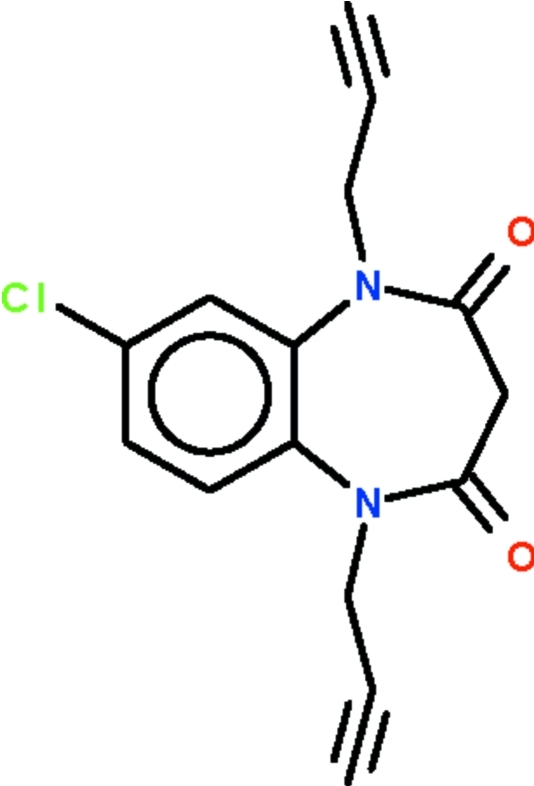


## Experimental

### 

#### Crystal data

C_15_H_11_ClN_2_O_2_
*M* *_r_* = 286.71
Monoclinic, 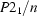

*a* = 10.7755 (3) Å
*b* = 7.6580 (2) Å
*c* = 16.7221 (5) Å
β = 103.621 (1)°
*V* = 1341.08 (7) Å^3^
*Z* = 4
Mo *K*α radiation
μ = 0.29 mm^−1^
*T* = 293 K
0.42 × 0.10 × 0.08 mm

#### Data collection

Bruker X8 APEXII diffractometer
Absorption correction: multi-scan (*SADABS*; Sheldrick, 1996 ▶) *T*_min_ = 0.889, *T*_max_ = 0.977
17112 measured reflections
3359 independent reflections
2679 reflections with *I* > 2σ(*I*)
*R*_int_ = 0.036

#### Refinement

*R*[*F* ^2^ > 2σ(*F* ^2^)] = 0.058
*wR*(*F* ^2^) = 0.172
*S* = 1.07
3359 reflections
189 parameters
2 restraints
H atoms treated by a mixture of independent and constrained refinement
Δρ_max_ = 1.36 e Å^−3^
Δρ_min_ = −0.55 e Å^−3^

### 

Data collection: *APEX2* (Bruker, 2007 ▶); cell refinement: *SAINT* (Bruker, 2007 ▶); data reduction: *SAINT*; program(s) used to solve structure: *SHELXS97* (Sheldrick, 2008 ▶); program(s) used to refine structure: *SHELXL97* (Sheldrick, 2008 ▶); molecular graphics: *X-SEED* (Barbour, 2001 ▶); software used to prepare material for publication: *publCIF* (Westrip, 2010 ▶).

## Supplementary Material

Crystal structure: contains datablocks global, I. DOI: 10.1107/S1600536810047008/nk2074sup1.cif

Structure factors: contains datablocks I. DOI: 10.1107/S1600536810047008/nk2074Isup2.hkl

Additional supplementary materials:  crystallographic information; 3D view; checkCIF report

## Figures and Tables

**Table 1:** Hydrogen-bond geometry (Å, °)

*D*—H⋯*A*	*D*—H	H⋯*A*	*D*⋯*A*	*D*—H⋯*A*
C9—H9⋯O1^i^	0.95 (3)	2.24 (3)	3.176 (3)	171 (3)

Symmetry code: (i) 
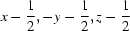
.

**Fig. 1.**
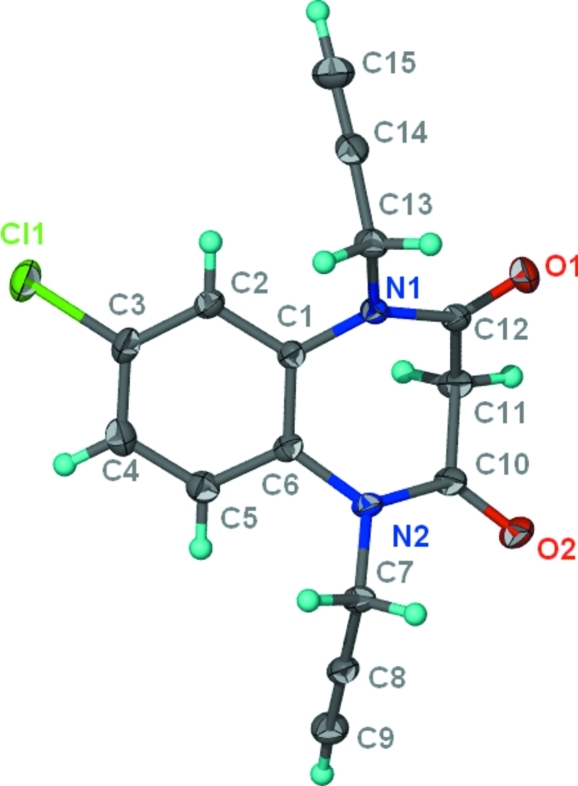
Displacement ellipsoid plot of C15H11ClN2O2 at the 50% probability level; hydrogen atoms are drawn as spheres of arbitrary radius.

**Fig. 2.**
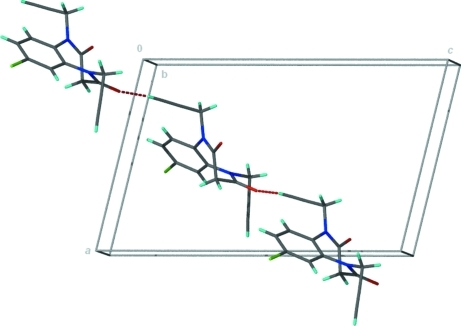
The hydrogen-bonded chain structure.

## supplementary crystallographic information

### Comment

We have reported the alkylation of 1,5-benzodiazepine-2,4-dione by alkylating agents in the presence of tetra-*n*-butylammonium bromide as catalyst (Mondieig *et al.*, 2005). In the present study, the amino H atoms are replaced by propargyl groups in the substituted 1,5-benzodiazepin-2,4-dione. The seven-membered ring of C_15_H_11_ClN_2_O_2_ (Scheme I, Fig. 1) adopts a boat-shaped conformation (with the C atoms of the fused-ring as the stern and the methylene C atom as the prow). The nitrogen atoms exists in a trigonal-planar coordination; one of the acetylenic H atoms forms a C–H···O hydrogen bond to the oxygen atom of an adjacent molecule to generate a linear chain (Fig. 2).

### Experimental

To a solution of the 7-chloro-1,5-benzodiazepine-2,4-dione (0.5 g, 2.38 mmol) in DMF (15 ml) was added potassium carbonate (0.98 g, 7.14 mmol), propargyl bromide (0.45 ml, 5.24 mmol) and tetra-*n*-butylammonium bromide (0.007 g, 0.25 mmol). Stirring was continued under reflux and the reaction was monitored by thin layer chromatography. On completion of the reactin, the mixture was filtered and the solvent removed under reduced pressure. The residue was separated by chromatography on a column of silica gel with ethyl acetate-hexane (1:1) as eluent. Yellow crystals were isolated when the solvent was allowed to evaporate.

### Refinement

Carbon-bound H-atoms were placed in calculated positions (C—H 0.93–0.97 Å) and were included in the refinement in the riding model approximation, with *U*(H) set to 1.2–1.5*U*_eq_(C). The final difference Fourier map had a peak in the vicinity of H4, and is 1.51 Å from C4. Attempts to treat this peak as a disorder component of the chlorine atom were unsuccessful. Furthermore, lowering to 2θ limit to 50 ° lead to a peak that has only 1 e Å^-3^ only.

### Crystal data

**Table:**

C_15_H_11_ClN_2_O_2_	*F*(000) = 592
*M**_r_* = 286.71	*D*_x_ = 1.420 Mg m^−^^3^
Monoclinic, *P*2_1_/*n*	Mo *K*α radiation, λ = 0.71073 Å
Hall symbol: -P 2yn	Cell parameters from 4883 reflections
*a* = 10.7755 (3) Å	θ = 2.5–28.0°
*b* = 7.6580 (2) Å	µ = 0.29 mm^−^^1^
*c* = 16.7221 (5) Å	*T* = 293 K
β = 103.621 (1)°	Prism, yellow
*V* = 1341.08 (7) Å^3^	0.42 × 0.10 × 0.08 mm
*Z* = 4	

### Data collection

**Table:**

Bruker X8 APEXII diffractometer	3359 independent reflections
Radiation source: fine-focus sealed tube	2679 reflections with *I* > 2σ(*I*)
graphite	*R*_int_ = 0.036
φ and ω scans	θ_max_ = 28.6°, θ_min_ = 2.1°
Absorption correction: multi-scan (*SADABS*; Sheldrick, 1996)	*h* = −14→14
*T*_min_ = 0.889, *T*_max_ = 0.977	*k* = −10→10
17112 measured reflections	*l* = −22→22

### Refinement

**Table:**

Refinement on *F*^2^	Primary atom site location: structure-invariant direct methods
Least-squares matrix: full	Secondary atom site location: difference Fourier map
*R*[*F*^2^ > 2σ(*F*^2^)] = 0.058	Hydrogen site location: inferred from neighbouring sites
*wR*(*F*^2^) = 0.172	H atoms treated by a mixture of independent and constrained refinement
*S* = 1.07	*w* = 1/[σ^2^(*F*_o_^2^) + (0.0732*P*)^2^ + 1.9878*P*] where *P* = (*F*_o_^2^ + 2*F*_c_^2^)/3
3359 reflections	(Δ/σ)_max_ = 0.001
189 parameters	Δρ_max_ = 1.36 e Å^−^^3^
2 restraints	Δρ_min_ = −0.55 e Å^−^^3^

### Fractional atomic coordinates and isotropic or equivalent isotropic displacement parameters (Å^2^)

**Table:**

	*x*	*y*	*z*	*U*_iso_*/*U*_eq_	
Cl1	0.57023 (7)	0.66801 (10)	0.13231 (5)	0.0407 (2)	
O1	0.6840 (2)	0.0626 (3)	0.47422 (12)	0.0409 (5)	
O2	0.44544 (18)	−0.2487 (2)	0.33490 (13)	0.0369 (5)	
N1	0.59018 (19)	0.2396 (3)	0.36901 (12)	0.0224 (4)	
N2	0.41244 (18)	0.0024 (3)	0.26154 (12)	0.0222 (4)	
C1	0.5433 (2)	0.2716 (3)	0.28363 (14)	0.0206 (5)	
C2	0.5776 (2)	0.4280 (3)	0.25108 (15)	0.0245 (5)	
H2	0.6347	0.5038	0.2845	0.029*	
C3	0.5270 (2)	0.4702 (3)	0.16968 (16)	0.0275 (5)	
C4	0.4440 (3)	0.3598 (4)	0.11757 (16)	0.0309 (6)	
H4	0.4107	0.3895	0.0628	0.037*	
C5	0.4119 (2)	0.2030 (3)	0.14941 (16)	0.0270 (5)	
H5	0.3575	0.1262	0.1148	0.032*	
C6	0.4589 (2)	0.1576 (3)	0.23202 (14)	0.0210 (5)	
C7	0.2750 (2)	−0.0392 (3)	0.23355 (15)	0.0232 (5)	
H7A	0.2456	−0.0894	0.2790	0.028*	
H7B	0.2279	0.0682	0.2176	0.028*	
C8	0.2469 (2)	−0.1612 (3)	0.16395 (16)	0.0252 (5)	
C9	0.2219 (3)	−0.2596 (4)	0.10749 (17)	0.0323 (6)	
C10	0.4874 (2)	−0.1152 (3)	0.31164 (16)	0.0263 (5)	
C11	0.6273 (2)	−0.0655 (3)	0.33946 (18)	0.0304 (6)	
H11A	0.6770	−0.1651	0.3648	0.037*	
H11B	0.6595	−0.0288	0.2926	0.037*	
C12	0.6388 (2)	0.0817 (3)	0.40064 (16)	0.0273 (5)	
C13	0.6048 (2)	0.3883 (3)	0.42683 (16)	0.0272 (5)	
H13A	0.5387	0.4737	0.4057	0.033*	
H13B	0.5928	0.3471	0.4793	0.033*	
C14	0.7301 (3)	0.4731 (3)	0.43968 (16)	0.0298 (5)	
C15	0.8290 (3)	0.5466 (4)	0.4487 (2)	0.0416 (7)	
H9	0.204 (3)	−0.342 (4)	0.0641 (16)	0.050 (10)*	
H15	0.906 (2)	0.611 (4)	0.457 (2)	0.057 (11)*	

### Atomic displacement parameters (Å^2^)

**Table:**

	*U*^11^	*U*^22^	*U*^33^	*U*^12^	*U*^13^	*U*^23^
Cl1	0.0390 (4)	0.0351 (4)	0.0491 (4)	−0.0006 (3)	0.0124 (3)	0.0178 (3)
O1	0.0477 (12)	0.0307 (10)	0.0343 (10)	−0.0038 (9)	−0.0102 (9)	0.0106 (8)
O2	0.0341 (10)	0.0184 (9)	0.0518 (12)	−0.0044 (7)	−0.0027 (9)	0.0070 (8)
N1	0.0227 (9)	0.0176 (9)	0.0237 (10)	−0.0010 (7)	−0.0011 (7)	0.0009 (7)
N2	0.0183 (9)	0.0161 (9)	0.0303 (10)	−0.0016 (7)	0.0017 (8)	−0.0010 (8)
C1	0.0173 (10)	0.0184 (10)	0.0255 (11)	0.0020 (8)	0.0041 (8)	0.0019 (8)
C2	0.0220 (11)	0.0196 (11)	0.0312 (12)	−0.0015 (9)	0.0051 (9)	0.0020 (9)
C3	0.0253 (12)	0.0252 (12)	0.0346 (13)	0.0026 (9)	0.0121 (10)	0.0092 (10)
C4	0.0299 (13)	0.0353 (14)	0.0276 (12)	0.0053 (11)	0.0070 (10)	0.0063 (10)
C5	0.0267 (12)	0.0269 (12)	0.0269 (12)	0.0007 (10)	0.0054 (10)	−0.0025 (9)
C6	0.0197 (10)	0.0173 (10)	0.0267 (11)	0.0008 (8)	0.0068 (9)	−0.0005 (9)
C7	0.0183 (11)	0.0194 (11)	0.0310 (12)	0.0003 (8)	0.0039 (9)	−0.0027 (9)
C8	0.0212 (11)	0.0212 (11)	0.0321 (12)	−0.0026 (9)	0.0040 (9)	0.0006 (9)
C9	0.0345 (14)	0.0296 (14)	0.0319 (14)	−0.0037 (11)	0.0062 (11)	−0.0052 (11)
C10	0.0245 (11)	0.0154 (11)	0.0352 (13)	0.0015 (9)	−0.0007 (10)	−0.0015 (9)
C11	0.0223 (12)	0.0169 (11)	0.0466 (15)	0.0027 (9)	−0.0029 (10)	0.0005 (10)
C12	0.0225 (11)	0.0211 (12)	0.0337 (13)	−0.0020 (9)	−0.0025 (10)	0.0049 (10)
C13	0.0272 (12)	0.0247 (12)	0.0276 (12)	−0.0006 (10)	0.0022 (10)	−0.0039 (10)
C14	0.0353 (14)	0.0217 (12)	0.0287 (12)	0.0010 (10)	0.0001 (10)	−0.0026 (10)
C15	0.0342 (15)	0.0341 (15)	0.0510 (18)	−0.0059 (12)	−0.0007 (13)	−0.0018 (13)

### Geometric parameters (Å, °)

**Table:**

Cl1—C3	1.743 (3)	C5—C6	1.399 (3)
O1—C12	1.221 (3)	C5—H5	0.9300
O2—C10	1.219 (3)	C7—C8	1.467 (3)
N1—C12	1.373 (3)	C7—H7A	0.9700
N1—C1	1.419 (3)	C7—H7B	0.9700
N1—C13	1.478 (3)	C8—C9	1.188 (4)
N2—C10	1.359 (3)	C9—H9	0.95 (3)
N2—C6	1.422 (3)	C10—C11	1.517 (3)
N2—C7	1.479 (3)	C11—C12	1.508 (4)
C1—C2	1.401 (3)	C11—H11A	0.9700
C1—C6	1.402 (3)	C11—H11B	0.9700
C2—C3	1.380 (3)	C13—C14	1.468 (4)
C2—H2	0.9300	C13—H13A	0.9700
C3—C4	1.381 (4)	C13—H13B	0.9700
C4—C5	1.390 (4)	C14—C15	1.183 (4)
C4—H4	0.9300	C15—H15	0.95 (3)
			
C12—N1—C1	123.5 (2)	N2—C7—H7A	109.0
C12—N1—C13	117.0 (2)	C8—C7—H7B	109.0
C1—N1—C13	118.91 (19)	N2—C7—H7B	109.0
C10—N2—C6	124.11 (19)	H7A—C7—H7B	107.8
C10—N2—C7	117.3 (2)	C9—C8—C7	178.9 (3)
C6—N2—C7	118.57 (19)	C8—C9—H9	177 (2)
C2—C1—C6	119.1 (2)	O2—C10—N2	122.8 (2)
C2—C1—N1	118.3 (2)	O2—C10—C11	121.9 (2)
C6—C1—N1	122.5 (2)	N2—C10—C11	115.2 (2)
C3—C2—C1	120.3 (2)	C12—C11—C10	108.2 (2)
C3—C2—H2	119.9	C12—C11—H11A	110.1
C1—C2—H2	119.9	C10—C11—H11A	110.1
C2—C3—C4	121.7 (2)	C12—C11—H11B	110.1
C2—C3—Cl1	118.7 (2)	C10—C11—H11B	110.1
C4—C3—Cl1	119.6 (2)	H11A—C11—H11B	108.4
C3—C4—C5	118.0 (2)	O1—C12—N1	121.3 (2)
C3—C4—H4	121.0	O1—C12—C11	122.9 (2)
C5—C4—H4	121.0	N1—C12—C11	115.7 (2)
C4—C5—C6	121.9 (2)	C14—C13—N1	112.9 (2)
C4—C5—H5	119.1	C14—C13—H13A	109.0
C6—C5—H5	119.1	N1—C13—H13A	109.0
C5—C6—C1	119.0 (2)	C14—C13—H13B	109.0
C5—C6—N2	118.4 (2)	N1—C13—H13B	109.0
C1—C6—N2	122.5 (2)	H13A—C13—H13B	107.8
C8—C7—N2	113.11 (19)	C15—C14—C13	177.7 (3)
C8—C7—H7A	109.0	C14—C15—H15	177 (2)
			
C12—N1—C1—C2	−136.1 (2)	C10—N2—C6—C1	−47.3 (3)
C13—N1—C1—C2	35.0 (3)	C7—N2—C6—C1	135.5 (2)
C12—N1—C1—C6	47.5 (3)	C10—N2—C7—C8	−80.8 (3)
C13—N1—C1—C6	−141.4 (2)	C6—N2—C7—C8	96.6 (2)
C6—C1—C2—C3	1.1 (3)	C6—N2—C10—O2	−178.7 (2)
N1—C1—C2—C3	−175.4 (2)	C7—N2—C10—O2	−1.5 (4)
C1—C2—C3—C4	−1.4 (4)	C6—N2—C10—C11	3.5 (3)
C1—C2—C3—Cl1	178.89 (18)	C7—N2—C10—C11	−179.3 (2)
C2—C3—C4—C5	0.1 (4)	O2—C10—C11—C12	−106.1 (3)
Cl1—C3—C4—C5	179.86 (19)	N2—C10—C11—C12	71.8 (3)
C3—C4—C5—C6	1.4 (4)	C1—N1—C12—O1	176.3 (2)
C4—C5—C6—C1	−1.6 (4)	C13—N1—C12—O1	4.9 (4)
C4—C5—C6—N2	174.4 (2)	C1—N1—C12—C11	−6.2 (3)
C2—C1—C6—C5	0.3 (3)	C13—N1—C12—C11	−177.5 (2)
N1—C1—C6—C5	176.7 (2)	C10—C11—C12—O1	107.6 (3)
C2—C1—C6—N2	−175.5 (2)	C10—C11—C12—N1	−69.9 (3)
N1—C1—C6—N2	0.9 (3)	C12—N1—C13—C14	83.2 (3)
C10—N2—C6—C5	136.9 (2)	C1—N1—C13—C14	−88.6 (3)
C7—N2—C6—C5	−40.3 (3)		

### Hydrogen-bond geometry (Å, °)

**Table:**

*D*—H···*A*	*D*—H	H···*A*	*D*···*A*	*D*—H···*A*
C9—H9···O1^i^	0.95 (3)	2.24 (3)	3.176 (3)	171 (3)

Symmetry codes: (i) *x*−1/2, −*y*−1/2, *z*−1/2.
